# Protease inhibitors, inflammatory markers, and their association with outcome in dogs with naturally occurring acute pancreatitis

**DOI:** 10.1111/jvim.15895

**Published:** 2020-09-07

**Authors:** Sharon Kuzi, Michal Mazaki‐Tovi, Jan S. Suchodolski, Dar Rimer, Jonathan A. Lidbury, Joerg M. Steiner, Agostino Buono, Ran Nivy, Gilad Segev, Itamar Aroch

**Affiliations:** ^1^ Department of Small Animal Internal Medicine The Hebrew University Veterinary Teaching Hospital and Koret School of Veterinary Medicine, Hebrew University of Jerusalem Rehovot Israel; ^2^ Gastrointestinal Laboratory, Texas A&M University College Station Texas USA

**Keywords:** antithrombin, canine, C‐reactive protein, cytokine, interleukin, α_1_‐proteinase inhibitor, α_2_‐antiplasmin

## Abstract

**Background:**

Acute pancreatitis (AP) presumably is associated with pancreatic protease activation, protease inhibitor (PI) depletion, and inflammatory mediator secretion.

**Objectives:**

Examine PIs and inflammatory mediator concentrations in dogs with AP and their association with death.

**Animals:**

Thirty‐one dogs diagnosed with AP based on clinical signs, ultrasonographic findings, and increased canine pancreatic lipase immunoreactivity (cPLI) and 51 healthy control dogs.

**Methods:**

Antithrombin and α_2_‐antiplasmin activity (ATA and α_2_AP, respectively) and concentrations of α_1_‐proteinase inhibitor (α_1_PI), α_2_‐macroglobulin (α_2_MG), C‐reactive protein (CRP), interleukins (ILs)‐2,6,8 and tumor necrosis factor‐α (TNF‐α) were prospectively measured. Severity of AP was assessed by clinical severity scoring systems.

**Results:**

Mortality rate was 19%. Antithrombin activity was lower (*P* = .004) and maximal CRP, IL‐6, and TNF‐α concentrations higher (*P* < .04) in the AP group compared to the controls, whereas IL‐2, IL‐8, α_1_PI, and α_2_AP concentrations did not differ between groups. Serum α_2_MG concentration was not reliably detected. Serum cPLI, CRP, and IL‐6 concentrations were significantly and positively correlated. The ATA was lower (*P* = .04), and canine acute pancreatitis severity (CAPS) scores higher (*P* = .009) in nonsurvivors compared to survivors. Higher CAPS scores were associated (*P <* .05) with decreased ATA and increased cPLI, CRP, and IL‐6 concentrations.

**Conclusions and Clinical Importance:**

Systemic inflammation in dogs with AP is manifested by increased inflammatory mediator concentrations, correlating with cPLI and CRP concentrations. Hypoantithrombinemia is associated with death. Serum concentrations of α_2_AP and α_1_PI are less useful prognostic markers. The CAPS score is a useful prognostic marker in dogs with AP.

**Abbreviations**
AP: acute pancreatitis
APP: acute phase protein
ATA: antithrombin activity
cPLI: canine pancreatic lipase immunoreactivity
CRP: C‐reactive protein
CSI: clinical severity index
D‐D: D‐dimer
DGGR‐lipase: 1,2‐o‐dilauryl‐rac‐glycero glutaric acid‐(6′‐methylresorufin) ester lipase
DIC: disseminated intravascular coagulation
ELISA: enzyme linked immunosorbent assay
FDR: false discovery rate
FFP: fresh frozen plasma
iCa: ionized (free) calcium
IL: interleukin
MODS: multiple organ dysfunction syndrome
PI: protease inhibitor
RI: reference interval
SIRS: systemic inflammatory response syndrome
TNF‐α: tumor necrosis factor‐α
α_1_PI: α1‐proteinase inhibitor
α_2_AP: α2‐antiplasmin
α2MG: α2‐macroglobulin
αMGs: α‐macroglobulins

## 1 INTRODUCTION

Acute pancreatitis (AP) is common in dogs and may be severe, with local and systemic complications and mortality rates ranging from 27% to 58%.^1^, ^2^, ^3^ The pathogenesis of AP may involve intra‐acinar cellular trypsinogen activation to trypsin, which can activate pancreatic proteases and lipases, resulting in cellular damage, tissue necrosis and inflammatory mediator secretion.^4^, ^5^ Recently however this common channel theory has been challenged by studies in mice, showing that intra‐acinar cellular trypsin activation is not the sole key factor in the pathogenesis of AP.^6^, ^7^

The inflammatory response in AP is complex, and may be localized or systemic, determining disease severity and course.^4^, ^5^, ^8^ Various inflammatory cytokines are secreted, among which tumor necrosis factor‐α (TNF‐α) and interleukin (IL)‐1β, IL‐2, and IL‐6 increase early,^4^, ^8^, ^9^, ^10^, ^11^, ^12^, ^13^, ^14^, ^15^ promoting leukocyte recruitment and inflammation and potentially leading to development of systemic inflammatory response syndrome (SIRS) and multiple organ dysfunction syndrome (MODS).^4^, ^8^, ^9^, ^10^, ^11^, ^12^, ^13^, ^14^, ^15^ In humans with AP, plasma cytokine concentrations are positively correlated with disease severity and prognosis.^12^, ^15^ Only 2 studies have characterized the inflammatory response in spontaneous AP in dogs.^16^, ^17^ In 1 study, serum TNF‐α concentration was not associated with disease severity.^16^ The second study showed decreased serum IL‐1β concentration when clinical signs had resolved, whereas IL‐6, IL‐10, IL‐18, and TNF‐α concentrations did not differ between survivors and nonsurvivors.^17^ The cytokine response patterns and their associations with the etiology, prognosis and response to treatment in dogs with AP are mostly unknown.

Many intra‐ and extra‐pancreatic defense mechanisms are responsible for pancreatic enzyme inhibition.^4^, ^5^ These include protease inhibitors (PIs), consisting of α‐macroglobulins (αMGs), α_1_‐proteinase inhibitor (α_1_PI, formerly named α_1_‐antitrypsin), antithrombin, and α_2_‐antiplasmin (α_2_AP).^4^, ^5^, ^18^, ^19^, ^20^, ^21^ The common channel theory proposes that AP ensues when defense mechanisms are overwhelmed, allowing ongoing enzyme‐induced tissue damage and resulting in inflammation.^5^ It is therefore to be expected that in severe AP, serum PI concentrations will be decreased, while pancreatic protease and lipase activities will be increased. If so, serum PI concentrations might serve as markers of severity and prognosis of AP,^4^, ^5^, ^18^, ^19^, ^20^ potentially guiding treatment (ie, administering fresh frozen plasma [FFP] to supplement PIs). Nevertheless, although several of these marker candidates showed promising associations with the severity of AP in humans, reported data are conflicting, with differences among studies in optimal sampling time points, short half‐life of some markers (eg, αMGs‐trypsin complexes), various tissue protease sources (eg, neutrophil polymorphonuclear elastase) and assay differences.^18^, ^22^ In dogs, α_2_MG concentration was significantly decreased in spontaneous AP compared to healthy controls, but was a poor disease severity indicator and was only weakly negatively correlated (*r* = −.147) with serum trypsin‐like immunoreactivity concentration.^23^ In another study of dogs with various diseases, α_1_PI concentration was normal, or even increased, in AP cases,^24^ in contrast to the hypothesized trend. Conversely, antithrombin activity (ATA) was decreased in dogs with AP, suggesting consumption.^25^

The primary aims of our prospective study were: (1) characterize the concentrations of several PIs (ie, ATA, α_2_MG, α_1_PI, and α_2_AP) and proinflammatory cytokines (ie, IL‐2, IL‐6, IL‐8, and TNF‐α) in dogs diagnosed with AP and in healthy control dogs and (2) investigate the associations of these analytes with short‐term death in dogs with AP. A secondary aim was to evaluate previously reported clinical severity scoring systems for AP in dogs, including local and systemic complications (ie, SIRS and hemostatic derangements), and their association with concentrations of PIs and inflammatory mediators and death.

## 2 MATERIALS AND METHODS

### 2.1 Selection of dogs

This prospective study was conducted from April 2015 to April 2016 in a university teaching hospital, which admits both referral and primary cases. It was approved by the Institutional Ethics Committee and included dogs with their owners' informed consent. Dogs diagnosed with AP were included in the study if they had no previous history of AP and presented with ≥2 of the following acute (duration ≤10 days) clinical signs: anorexia, vomiting, diarrhea and abdominal pain. Diagnosis of AP was confirmed by 1,2‐o‐dilauryl‐rac‐glycero glutaric acid‐(6′‐methylresorufin) ester (DGGR) lipase activity >250 U/L (reference interval [RI], 5‐107 U/L), or by serum amylase activity ≥4500 U/L (above approximately 3× −fold the amylase upper reference limit), along with ultrasonographic evidence of AP, including ≥2 of the following: pancreatomegaly, pancreatic hypo‐echogenicity, peripancreatic hyperechoic mesentery, and presence of peripancreatic fluid.^26^ Additionally, canine pancreatic lipase immunoreactivity (cPLI, [canine Spec cPL], Idexx Laboratories, Westbrook, Maine) concentration was measured, and only dogs in which cPLI concentration was ≥400 μg/L (RI, 0‐200 μg/L), either at presentation or on the next day, thereby in agreement with increased DGGR lipase or amylase activity, were included. Dogs that died within 24 hours after presentation, and those for which owners had elected euthanasia for financial reasons were excluded. Finally, dogs with a known chronic underlying disease, or dogs in which AP was considered a minor disease process (eg, AP in a dog with immune‐mediated hemolytic anemia) were excluded.

Staff‐ and breeder‐owned dogs were included in the healthy control group. They were deemed healthy based on normal history, physical examination findings, CBC, serum biochemistry, cPLI concentration, and C‐reactive protein (CRP) concentration (RI, 0‐7 mg/L).

### 2.2 PIs, proinflammatory cytokines, hemostatic tests, and CRP measurement

Blood samples were collected at presentation (day 0) and 24 hours post‐presentation (day 1). Complete blood count (Advia 2120i, Siemens Medical Solutions Diagnostics GmbH, Erlangen, Germany), routine serum biochemistry and DGGR‐lipase activity (Cobas 6000, Roche, Mannheim, Germany, at 37°C) were measured at presentation in all dogs. To investigate whether ATA and α_2_AP activity changed because of their anticoagulant or PI role or both, hemostatic tests were performed, including prothrombin time (PT), activated partial thromboplastin time (aPTT; ACL‐9000, Instrumentation Laboratory, Milano, Italy) and D‐dimer (D‐D) concentration (Cobas 6000, Roche, Mannheim, Germany, at 37°C; Reagent, Tina‐quant D‐Dimer Gen.2, Roche, Mannheim, Germany). If all other hemostatic tests were within RI, major antithrombin and α_2_AP consumption because of their anticoagulant activity was considered less likely. Serum and plasma sample aliquots from days 0 and 1 were stored for up to 1 year at −80°C, pending measurement of cPLI, CRP (Tridelta Development Ltd., Maynooth, Ireland), ATA and α_2_AP activities (ACL‐9000, Instrumentation Laboratory, Milano, Italy), α_2_MG (Human α_2_MG DuoSet ELISA R&D Systems, DY1938, Minneapolis, MN), α_1_PI (in‐house ELISA, Gastrointestinal Laboratory, Texas A&M University, College Station, Texas), and IL‐2, IL‐6, IL‐8, and TNF‐α (Canine Proinflammatory Panel 3, Meso Scale Discovery, Meso Scale Diagnostics, Rockville, Maryland; Analyzer, MSD Quickplex SQ 120, Meso Scale Diagnostics, Rockville, Maryland; Analysis software, DISCOVERY WORKBENCH 4.0, Meso Scale Diagnostics, Rockville, Maryland). Samples requiring analysis in a different laboratory (CRP, cPLI, α_1_PI, IL‐2, IL‐6, IL‐8, and TNF‐α) were shipped on dry ice at the end of the study. Treatments were implemented at the discretion of the attending clinicians. Clinicians were blinded to the PI and cytokine concentrations. Demographic, historical, clinical, laboratory, imaging, and outcome (including duration of hospitalization) data were collected from the medical records.

Serum IL‐2, IL‐6, IL‐8, and TNF‐α concentrations were measured using a multiplex electrochemiluminescence immunoassay previously used in dogs according to the manufacturers' instructions.^27^, ^28^, ^29^ The minimal and maximal detection limits reported by the manufacturer for IL‐2, IL‐6, IL‐8, and TNF‐α were 7.6 and 20 000, 2.4 and 10 000, 1.3 and 10 000, and 0.17 and 5000 pg/mL, respectively. The α_2_MG concentration was measured using a human‐specific enzyme linked immunosorbent assay (ELISA),^30^ with a dynamic range of 0.625 to 40.0 ng/mL, and intra‐assay variance coefficient of <5%. The use of this assay had not been reported previously in dogs.

### 2.3 Clinical scoring indexes for AP and AP‐associated local and systemic complications

Clinical disease severity scores were assigned to each dog with AP using 3 previously published canine AP clinical scoring indexes (CSIs),^31^, ^32^ including a recently validated organ score CSI,^31^, ^33^ the canine acute pancreatitis severity (CAPS) and the simplified CAPS (sCAPS^32^; Appendix S1).

Local abdominal complications included peripancreatic or diffuse effusion, cholestasis, and intestinal ileus.^31^, ^33^ Systemic complications included: (1) acute kidney injury (AKI), diagnosed based on the International Renal Interest Society grading system^34^; (2) disseminated intravascular coagulation (DIC), defined as presence of anomalies in each of the following categories: coagulation factor and platelet consumption (≥2 of the following: thrombocytopenia [platelet count, <143 × 10^9^/L], prolonged aPTT [aPTT, >20 s; RI, 17.4‐11.0 s] and PT [PT, >10 s; RI, 8.4‐6.0 s]), coagulation factor inhibitor consumption (hypoantithrombinemia [ATA <87%; RI, 87‐140%]) and increased fibrinolysis (D‐D concentration > 500 ng/mL; RI, <250 ng/mL)^35^; (3) Hypercoagulability (suspected when PT, aPTT and platelet count were all within RI, while D‐D concentration was increased, or with presence of clinical or imaging evidence of thrombosis [ie, absent femoral pulse in absence of hypotension, Doppler‐ultrasonography evidence of a thrombus interrupting blood flow])^36^; and (4) SIRS diagnosed based on criteria used in the CAPS scoring system^32^ (Appendix S1). Diabetic ketoacidosis (DKA) was diagnosed based on presence of persistent fasting hyperglycemia (blood glucose concentration >250 mg/dL), acidosis (venous blood pH <7.34) and hyperketonemia (serum β‐hydroxybutyric acid [BHBA; Cobas 6000, Roche, Mannheim, Germany] concentration ≥0.8 mmol/L; RI, 0.0‐0.7 mmol/L).^37^

Survivors were defined as dogs alive at 30 days post‐presentation, whereas nonsurvivors consisted of dogs that died or were euthanized because of AP‐related clinical deterioration within 30 days of presentation. All deaths were AP‐related.

### 2.4 Statistical analysis

The distribution pattern of continuous variables was examined using the Shapiro‐Wilk's test. Pearson's or Spearman's correlation tests, based on data distribution pattern, were used to examine relationships between continuous variable pairs. Results of most variables were distributed non‐normally, and with the limited study group sizes, group continuous variables were compared using Mann‐Whitney *U*‐tests. Associations between categorical variable pairs were examined using Fisher's exact tests. Because the control group's PI and inflammatory mediator concentrations were measured only once, but measured twice in the AP group, several comparisons were made. The lowest ATA and α_2_AP activity, the highest CRP and cytokine concentrations and both lowest and highest α_1_PI concentrations of day 0 and day 1 measurements of dogs with AP were used for study group comparisons, in addition to comparisons of the control group's single measurements with the study group's day 0 and day 1 measurements. Only analytes that differed significantly between dogs with AP and healthy control dogs were further tested for associations with outcome in dogs with AP. All tests were 2‐tailed. Raw *P* values ≤.05 were adjusted for false discovery rate (FDR) using the Benjamini‐Hochberg procedure.^38^ In such cases, both the unadjusted and adjusted *P* values are presented in the tables. Throughout the text, adjusted *P* values are presented, unless otherwise specified. Statistical analyses were performed using a statistical software package (spss 25.0 for Windows, IBM, Armonk, NY).

## 3 RESULTS

Our study initially included 33 dogs with AP and 51 healthy control dogs. Two dogs in which serum DGGR lipase activity was increased (>2000 U/L in both), but day 0 and day 1 serum cPLI concentrations were <400 μg/L, were excluded. Mortality rate in the remaining 31 dogs with AP was 19% (6 nonsurvivors). Nonsurvivor dogs died within 3‐14 days from presentation (median, 5 days). The age of dogs with AP (median, 9 years; range, 3‐15 years) was higher (*P* = .02) compared to that of the control group (median, 4.5 years; range, 0.75‐13). The AP group included 13 females (42%; neutered, 12) and 18 males (58%; neutered, 11). The control group included 32 females (63%; neutered, 20), and 19 males (37%; neutered, 13). Most dogs in the AP and control groups were of mixed breed (18; 58% and 20; 39%, respectively). No sex difference was found between groups (unadjusted *P* = .11).

### 3.1 PIs and proinflammatory cytokines in the AP and control groups

Day 1 and minimal ATA measured during the observation period were significantly (*P = .03 and P = .004*, respectively) lower in dogs with AP compared to controls (Table 1). Serum α_2_AP activity did not differ between groups. Peak α_1_PI concentration was higher (unadjusted *P* = .05) in the AP group, but insignificantly so after adjusting for FDR (*P* = .1; Table 1). Day 0, day 1, and maximal serum CRP and IL‐6 concentrations were significantly (*P* = .004 for all) higher in dogs with AP compared to the control group. Maximal TNF‐α concentration was higher in the AP group (*P* = .03) compared to the control group, whereas IL‐8 and IL‐2 concentrations did not differ between these 2 groups (Table 2).

**TABLE 1:** Protease inhibitor concentrations in 31 dogs with acute pancreatitis and in 51 healthy control dogs

Analyte		Controls^a^ (n = 51)	Acute pancreatitis (n = 31)	*P* value	Adjusted *P* value^b^
Antithrombin activity_min_ (%)^c^	n (%) Median (range)	25 (49) 160 (116‐200)	25 (81) 119 (41‐193)	<.001	.004
Antithrombin activity_0_ (%)^c^	n (%) Median (range)		25 (81) 149 (41‐193)	.03	.09
Antithrombin activity_1_ (%)^c^	n (%) Median (range)		25 (81) 134 (63‐194)	.008	.03
α2‐Antiplasmin activity_min_ (%)	n (%) Median (range)	28 (55) 100 (90‐186)	25 (81) 99.0 (88‐106)	.44	
α2‐Antiplasmin activity_0_ (%)	n (%) Median (range)		25 (81) 102 (88‐108)	.42	
α2‐Antiplasmin activity_1_ (%)	n (%) Median (range)		25 (81) 100 (91‐106)	.64	
α1‐Proteinase inhibitor_min_ (mg/L)^d^	n (%) Median (range)	28 (55) 1801 (1337‐2366)	31 (100) 1701 (1390‐3251)	.44	
α1‐Proteinase inhibitor_max_ (mg/L)^d^	n (%) Median (range)		31 (100) 1974 (1475‐3286)	.05	.11
α1‐Proteinase inhibitor_0_ (mg/L)	n (%) Median (range)		31 (100) 2037 (1390‐3286)	.12	
α1‐Proteinase inhibitor_1_ (mg/L)	n (%) Median (range)		31 (100) 1788 (1400‐3251)	.98	

Abbreviation: n, number of cases.

^a^ Each analyte was measured once in the control group and twice in dogs with acute pancreatitis. Subscripts 0, 1, min and max refer to concentrations on day 0 (presentation), day 1 (1 day post‐presentation) and minimal or maximal concentrations during days 0 and 1, respectively.

^b^ Adjusted *P* value, significant raw *P* values were adjusted using the Benjamini‐Hochberg false discovery rate procedure.

^c^ Antithrombin activity reference interval is 87%<.

^d^ α1‐Proteinase inhibitor reference interval is 732‐1802 mg/L.

**TABLE 2:** Serum C‐reactive protein and cytokine concentrations in 31 dogs with acute pancreatitis and in 51 healthy control dogs

Analyte		Controls^a^ (n = 51)	Acute pancreatitis (n = 31)	*P* value	Adjusted *P* value^b^
C‐reactive protein_0_ ^c^ (mg/L)	n (%) Median (range)	28 (55) 0.0 (0.0‐9.0)	29 (93.5) 100.8 (10.1‐614.4)	<.001	.004
C‐reactive protein_1_ (mg/L)	n (%) Median (range)		27 (87) 96.2 (7.2‐352.0)	<.001	.004
C‐reactive protein_max_ (mg/L)	n (%) Median (range)		31 (100) 103.3 (10.1‐614.4)	<.001	.004
IL‐2_0_ (pg/mL)	n (%) Median (range)	28 (55) 37.1 (7.7‐119.4)	29 (93.5) 27.4 (3.3‐375.3)	.43	
IL‐2_Day 1_ (pg/mL)	n (%) Median (range)		27 (87) 39.2 (4.1‐314.7)	.82	
IL‐2_max_ (pg/mL)	n (%) Median (range)		31 (100) 41.4 (5.6‐375.3)	.41	
IL‐6_0_ (pg/mL)	n (%) Median (range)	28 (55) 26.6 (10.3‐104.0)	29 (93.5) 106.4 (14.7‐1884.9)	<.001	.004
IL‐6_1_ (pg/mL)	n (%) Median (range)		27 (87) 90.3 (10.4‐946.3)	<.001	.004
IL‐6_max_ (pg/mL)	n (%) Median (range)		31 (100) 119.4 (14.7‐1884.9)	<.001	.004
IL‐8_0_ (pg/mL)	n (%) Median (range)	28 (55) 3166 (709‐7114)	29 (93.5) 3086.9 (289‐11 934.9)	.88	
IL‐8_1_ (pg/mL)	n (%) Median (range)		27 (87) 3260.6 (475.4‐11 644.4)	.37	
IL‐8_max_ (pg/mL)	n (%) Median (range)		31 (100) 5309 (475‐11 935)	.08	
TNF‐α_0_ (pg/mL)	n (%) Median (range)	28 (55) 5.7 (1.8‐43.6)	29 (93.5) 6.90 (0.56‐233.00)	.22	
TNF‐α_1_ (pg/mL)	n (%) Median (range)		27 (87) 5.8 (0.8‐51.4)	.23	
TNF‐α_max_ (pg/mL)	n (%) Median (range)		31 (100) 10.4 (0.8‐233.0)	.008	.03

Abbreviations: IL, interleukin; n, number of cases; TNF‐α, tumor necrosis factor‐α.

^a^ Each analyte was measured once in the control group and twice in dogs with acute pancreatitis; Subscripts 0, 1 and max refer to concentrations on day 0 (presentation), day 1 (1 day post‐presentation) and maximal concentrations during days 0 and 1, respectively.

^b^ Adjusted *P* value, significant raw *P* values were adjusted using the Benjamini‐Hochberg false discovery rate procedure.

^c^ C‐reactive protein reference interval is 0‐7 (mg/L).

Day 0 and day 1 α_2_MG concentrations in dogs with AP (day 0 median, 1.6 ng/mL; range, 0‐7.9; day 1 median, 1.5 ng/mL; range, 0‐7.7) as well as in healthy control dogs (median, 1.15 ng/mL; range, 0‐7.5) were extremely low, or undetectable, and were not different between groups.

### 3.2 Pancreatic lipase, PIs, proinflammatory cytokines, hemostatic tests, and outcome

The DGGR lipase activity and cPLI concentration at presentation were moderately, positively correlated (*r* = .662; *P =* .001). Day 0 DGGR‐lipase activity and day 1 cPLI concentration were higher, albeit insignificantly, among nonsurvivors compared to survivors (unadjusted *P* = .09 for both, Table 3). Hypoantithrombinemia on days 0 and 1 was significantly (*P* = .04 and *P* = .007, respectively) associated with death (Table 3), whereas serum concentrations of IL‐6, TNF‐α, and CRP were not associated with death (Table 4).

**TABLE 3:** Antithrombin activity, pancreatic lipase immunoreactivity concentration and lipase activity, and their association with the outcome in 31 dogs with acute pancreatitis

Analyte		Survivors (n = 25)	Nonsurvivors (n = 6)	*P* value	Adjusted *P* value^a^
ATA_0_ (%)	n (%) Median (range)	21 (84) 154 (41‐193)	5 (83) 91 (75‐140)	.015	.04
ATA_1_ (%)	n (%) Median (range)	21 (84) 131 (41‐193)	5 (83) 82 (63‐134)	.002	.007
ATA_min_ (%)	n (%) Median (range)	21 (84) 131 (41‐193)	5 (83) 77 (63‐111)	.003	.009
cPLI_0_ (μg/L)	n (%) Median (range)	24 (96) 2326.0 (536‐51 968)	6 (100) 4316 (1256‐13 896)	.16	
cPLI_1_ (μg/L)	n (%) Median (range)	22 (88) 1530 (123‐30 576)	6 (100) 4331 (1107‐26 352)	.09	
cPLI_max_ (μg/L)	n (%) Median (range)	25 (100) 2552 (536‐51 968)	6 (100) 4466 (1915‐26 352)	.11	
Lipase activity_0_ (U/L)	n (%) Median (range)	19 (76) 1232 (294‐13 085)	5 (83) 3000 (610‐7876)	.09	

*Notes:* cPLI reference interval is 0‐200 (μg/L); Lipase, 1,2‐o‐dilauryl‐rac‐glycero glutaric acid‐(6′‐methylresorufin) ester, (DGGR) lipase activity, DGGR lipase reference interval is 5‐107 (U/L); Subscripts 0, 1, min and max refer to concentrations on day 0 (presentation), day 1 (1 day post‐presentation) and minimal and maximal concentrations during days 0 and 1, respectively.

Abbreviations: ATA, antithrombin activity; cPLI, canine pancreatic‐like immunoreactivity; n, number of cases.

^a^ Adjusted *P* value, significant raw *P* values were adjusted using the Benjamini‐Hochberg false rate discovery procedure.

**TABLE 4:** Serum C‐reactive protein, interleukin‐6, and tumor necrosis factor‐α concentrations and their association with the outcome in 31 dogs with acute pancreatitis

Analyte		Survivors (n = 25)	Nonsurvivors (n = 6)	*P* value^a^
CRP_0_ (mg/L)	n (%) Median (range)	24 (96) 100 (10‐471)	6 (100) 122 (13‐614)	.68
CRP_1_ (mg/L)	n (%) Median (range)	22 (88) 65 (7‐230)	6 (100) 155 (25‐352)	.08
CRP_max_ (mg/L)	n (%) Median (range)	25 (100) 101 (10‐471)	6 (100) 174 (25‐614)	.35
IL‐6_0_ (pg/mL)	n (%) Median (range)	24 (96) 104 (15‐521)	6 (100) 209 (42‐1885)	.12
IL‐6_1_ (pg/mL)	n (%) Median (range)	22 (88) 69 (10‐946)	6 (100) 133 (61‐905)	.16
IL‐6_max_ (pg/mL)	n (%) Median (range)	25 (100) 115 (14.7‐946)	6 (100) 209 (65.3‐1885)	.21
TNF‐α_max_ (pg/mL)	n (%) Median (range)	25 (100) 8.05 (0.80‐233)	6 (100) 11.6 (3.6‐44.0)	.92

*Notes:* Subscripts 0, 1 and max refer to concentrations on day 0 (presentation), day 1 (1 day post‐ presentation) and maximal levels during days 0 and 1, respectively.

Abbreviations: CRP, C‐reactive protein; IL, interleukin; n, number of cases; TNF, tumor necrosis factor.

^a^ Raw *P* values; Because all comparisons depicted in this table were insignificant, all *P* values presented above are raw values, unadjusted for false discovery rate.

Day 0 cPLI concentration was strongly and positively correlated with day 1 serum IL‐6 and day 0 serum CRP concentrations (Table 5). Day 0 serum CRP concentration was positively and moderately correlated with day 1 serum IL‐6 and day 0 serum cholesterol and triglyceride concentrations (Table 5). Day 1 serum cPLI and CRP concentrations were inversely and moderately correlated with serum ionized calcium (iCa) concentration (Table 5). Day 0 and day 1 serum IL‐2, IL‐6, and IL‐8 concentrations were positively and strongly correlated (Table 5). The results of all hemostatic analytes were not associated with death, and all hemostatic test results, in both survivors and nonsurvivors of AP, were within RI (Table 6).

**TABLE 5:** Significant correlations (*r* > .5) between pancreatic lipases, protease inhibitors, hemostatic analytes, C‐reactive protein, and cytokines in 31 dogs with acute pancreatitis

Analyte	Correlated with	*r* ^a^	*P* value
cPLI_0_	IL‐6_1_	.75	0
	CRP_0_	.52	.005
cPLI_1_	IL‐6_0_	.55	.003
	IL‐6_1_	.68	0
	IL‐6_max_	.57	.001
	Ionized calcium	−.62	.009
CRP_0_	IL‐6_1_	.58	.001
	cholesterol	.68	0
	TG	.52	.023
CRP_1_	IL‐6_1_	.65	0
	IL‐6_max_	.51	.006
	Ionized calcium	−.67	.003
CRP_max_	IL‐6_1_	.58	.001
	IL‐6_max_	.62	0
	Ionized calcium	−.58	.009
	Cholesterol	.58	.001
IL‐2_0_	IL‐6_0_	.65	0
	IL‐6_max_	.56	.001
	IL‐8_0_	.92	0
	IL‐8_1_	.60	.001
	IL‐8_max_	.74	0
	TNFα_1_	.53	.004
IL‐2_1_	IL‐8_1_	.89	.000
	IL‐8_max_	.84	.000
	TNFα_0_	.504	.007
	TNFα_1_	.80	0
	TNFα_max_	.84	0
	Hemoglobin	−.52	.007
	Hematocrit	−.53	.005
IL‐2_max_	IL‐6_1_	.61	.001
	IL‐8_0_	.67	0
	IL‐8_1_	.89	0
	IL‐8_max_	.82	0
	TNFα_1_	.839	0
	Hematocrit	−.53	.003
IL‐6_1_	Ionized calcium	−.73	.001
	Amylase	.62	.001
IL‐8_0_	TNFα_1_	.60	.001
IL‐8_1_	TNFα_0_	.59	.001
	TNFα_1_	.86	.000
	TNFα_max_	.93	0
α_1_PI_0_	Duration of clinical signs	−.50	.002
α_2_antiplasmin_0_	Duration of clinical signs	−.52	.007

*Notes:* Subscripts 0, 1 max and min refer to concentrations on day 0 (presentation), day 1 (1 day post‐presentation), maximal and minimal concentrations during days 0 and 1, respectively.

Abbreviations: _α1_PI, α_1_‐proteinase inhibitor concentration; clinical signs duration, lag of time from onset of clinical signs to presentation to the hospital; cPLI, canine pancreatic‐like immunoreactivity; CRP, C‐reactive protein; IL, interleukin; TNFα, tumor necrosis factor α.

^a^ Spearman's correlation tests were done when ≥1 of the tested variables had non‐normal distribution, whereas Pearson's correlation tests were done when both tested variables were distributed normally.

**TABLE 6:** Hemostatic test results and the outcome of 31 dogs with acute pancreatitis

Analyte	Reference interval		Survivors (n = 25)	Nonsurvivors (n = 6)	*P* value	Adjusted *P* value^a^
Prothrombin time_0_ (s)	6.0‐8.4 (s)	n (%) Median (range)	20 (80) 7.1 (6.9‐10.5)	5 (83) 7.2 (7.0‐8.7)	.22	
Prothrombin time_1_ (s)		n (%) Median (range)	21 (84) 7.1 (6.8‐8.3)	5 (83) 7.4 (7.0‐9.4)	.04	.09
Prothrombin time_max_ (s)		n (%) Median (range)	21 (84) 7.1 (6.9‐10.5)	5 (83) 7.5 (7.0‐9.4)	.09	
aPTT_0_ (s)	11.0‐17.4 (s)	n (%) Median (range)	20 (80) 13.2 (8.7‐27.2)	5 (83) 11.9 (10.4‐75.0)	.37	
aPTT_1_ (s)		n (%) Median (range)	21 (84) 12.7 (7.5‐25.5)	5 (83) 12.9 (10.4‐34.2)	.53	
aPTT _max_ (s)		n (%) Median (range)	21 (84) 13.2 (8.7‐27.2)	5 (83) 12.9 (10.4‐75.0)	.95	
D‐dimer_0_ (ng/mL)	0‐250 (ng/mL)	n (%) Median (range)	20 (80) 192 (14‐4298)	4 (67) 157 (108‐875)	.79	
D‐dimer_1_ (ng/mL)		n (%) Median (range)	21 (84) 201 (88‐4034)	5 (83) 329 (69‐2223)	.75	
D‐dimer_max_ (ng/mL)		n (%) Median (range)	21 (80) 208 (88‐4298)	5 (83) 329 (108‐2223)	.70	

*Notes:* Subscripts 0, 1 max, and min refer to levels on day 0 (presentation), day 1 (1 day post presentation), and maximal and minimal concentrations during days 0 and 1, respectively.

Abbreviations: aPTT, activated partial thromboplastin time; n, number of cases; PT, prothrombin time.

^a^ Adjusted *P* value, significant raw *P* values were adjusted using the Benjamini‐Hochberg false discovery rate procedure.

### 3.3 CSI score associations with PI and proinflammatory cytokine concentrations and with outcome

Among the 3 applied AP CSIs, only the CAPS results were significantly (*P* = .009) associated with outcome (Table 7). The CAPS and sCAPS scores were strongly correlated (*r* = .89; *P <* .001), whereas the CAPS and organ score CSI scores were weakly correlated (*r =* .45; *P* = .01). The CAPS score associations with cPLI concentration and with PIs and inflammatory mediator concentrations that were significantly higher in dogs with AP compared to the control group and also associated with outcome of AP were examined. Higher (≥11) CAPS scores were significantly associated with decreased day 1 and minimal ATA, as well as with higher day 0 cPLI and day 1 CRP and IL‐6 concentrations (*P* = .01, *P* = .03, *P* = .03, *P* = .05, and *P* = .04, respectively).

**TABLE 7:** Association between results of clinical severity scoring systems for acute pancreatitis in dogs^a^ and the outcome of 31 dogs with acute pancreatitis

	Severe^b^ acute pancreatitis		Mild to moderate^c^ acute pancreatitis			
	Survivors (n = 25)	Nonsurvivors (n = 6)	Survivors (n = 25)	Nonsurvivors (n = 6)		
Scoring system	n (%)	n (%)	n (%)	n (%)	*P* value	Adjusted *P* value^d^
CAPS^e^	7 (28)	6 (100)	18 (72)	0 (0)	.002	.009
sCAPS^f^	7 (28)	5 (83)	18 (72)	1 (5)	.02	.06
Organ score	14 (56)	6 (100)	11 (44)	0 (0)	.05	.12

Abbreviation: n, number of cases.

^a^ (1) Based on Ruaux CG and Atwell RB. A severity score for spontaneous canine acute pancreatitis. Aust Vet J 1998;76:804‐808; (2) Based on Fabres V, Dossin O, Reif C, et al. Development and validation of a novel clinical scoring system for short‐term prediction of death in dogs with acute pancreatitis. J Vet Intern Med 2019;33:499‐507; (3) Based on Kuzi S, Mazor R, Segev G, et al. Prognostic markers and assessment of a previously published clinical severity index in 109 hospitalized dogs with acute presentation of pancreatitis. Vet Rec. 2019 Oct 29. pii: vetrec‐2019‐105 364. doi:10.1136/vr.105364. [Epub ahead of print].

^b^ Severe disease was assigned to dogs with CAPS score ≥ 11, sCAPS score ≥ 6, and an organ score ≥ 5.

^c^ Mild to moderate disease was assigned to dogs with a CAPS score ≤ 10, a sCAPS score ≤ 5, and an organ score ≤ 4.

^d^ Significant “raw” *P* values were adjusted using the Benjamini‐Hochberg procedure for false discovery rate.

^e^ Canine acute pancreatitis severity scoring, based on Fabres V, Dossin O, Reif C, et al. Development and validation of a novel clinical scoring system for short‐term prediction of death in dogs with acute pancreatitis. J Vet Intern Med. 2019;33:499‐507.

^f^ Simplified canine acute pancreatitis severity scoring, based on Fabres V, Dossin O, Reif C, et al. Development and validation of a novel clinical scoring system for short‐term prediction of death in dogs with acute pancreatitis. J Vet Intern Med. 2019;33:499‐507.

### 3.4 Associations of AP‐related complications and duration of clinical signs with PI and proinflammatory cytokine concentrations and with outcome

Several systemic complications recorded in the AP group were not associated with death. Disseminated intravascular coagulation was diagnosed in 1 nonsurvivor. Severe thrombocytopenia (platelets <63 000/μL) occurred in 2 dogs (1 nonsurvivor with DIC and 1 survivor). Hypercoagulability was suspected in 9 dogs (2 nonsurvivors). Acute kidney injury was diagnosed in 8 dogs (3 nonsurvivors). Systemic inflammatory response syndrome was noted in 24 dogs (77%; including all 6 nonsurvivors). Diabetic ketoacidosis was present in 5 dogs (1 nonsurvivor). Local abdominal complications were noted in 9 dogs (2 nonsurvivors). All of these specific complications were not associated with PI and proinflammatory cytokine concentrations.

The time lag from onset of clinical signs to presentation (median, 3 days; range, 0.5‐10 days) was not associated with death (*P* = .17). Hospitalization duration was shorter (*P* = .04) when dogs were presented earlier (duration of clinical signs ≤2 days; median, 4 days, range 2‐14) compared to dogs presented later (median, 5 days; range, 2‐17 days). The α_1_PI concentration was moderately and inversely correlated with the time lag from onset of clinical signs to presentation (Table 5) and was significantly (*P* = .008) higher in dogs presented early (median, 2208 mg/L; range, 1765‐3287) compared to those presented later (median, 1735 mg/L; range, 1390‐2641 mg/L).

## 4 DISCUSSION

The severity of AP presumably is determined by the extent of pancreatic protease and inflammatory mediator release into the systemic circulation, leading to both local and systemic complications.^4^, ^5^, ^11^, ^31^, ^32^ The latter include SIRS and MODS, and their manifestations contribute to the CSI scores, which have been associated with the prognosis of AP in both humans and dogs.^4^, ^5^, ^11^, ^31^, ^32^, ^33^ In our study, concentrations of pancreatic lipase (cPLI and DGGR lipase) and inflammatory cytokines did not differ between survivors and nonsurvivors of AP. The former finding is in disagreement with previous studies of AP in dogs, in which markedly increased cPLI concentrations^39^ or serum lipase activities^40^ were more frequent in nonsurvivors. This discrepancy possibly resulted from differences in duration of clinical signs before presentation, reflected in higher (albeit insignificantly so) day 1 cPLI concentrations among nonsurvivors in our study. In addition, in our study, higher CAPS scores (≥11), reflective of more severe AP, were associated with cPLI concentration, supporting results of the aforementioned studies.^39^, ^40^ In our study, serum cPLI concentration was significantly and positively correlated with serum inflammatory marker concentrations, including IL‐6 and CRP, linking increased cPLI concentration with the inflammatory response. The lack of association between inflammatory cytokine concentrations and outcome in our study is consistent with previous findings, suggesting that inflammatory cytokines are not useful prognostic markers in dogs with AP.^17^ Nevertheless, the utility of serial measurements in clinical decision making warrants future evaluation.

In agreement with previous findings,^25^, ^41^ ATA was significantly lower in nonsurvivors, decreasing early during hospitalization. The activity of α_2_AP was examined in dogs with AP for the first time in our study. Its activity in the AP and control groups was similar, suggesting a limited role of α_2_AP in dogs with AP.

In contrast to antithrombin, which is depleted in dogs with AP, α_1_PI concentration neither differed between controls and dogs with AP nor between survivors and nonsurvivors of AP. Alpha‐1 proteinase inhibitor is considered the main plasma pancreatic protease inhibitor,^21^, ^42^ and therefore is expected to be consumed, and possibly depleted during AP. However, contrary to this hypothesis, α_1_PI is not depleted, but rather is increased in humans with AP,^22^, ^43^, ^44^ supporting a positive acute phase protein (APP) role, beyond its PI function.^22^, ^43^, ^44^, ^45^ The APP role of α_1_PI in dogs is controversial. Most studies demonstrate increased serum α_1_PI concentration in dogs with inflammatory diseases,^24^, ^46^, ^47^ although in 1 study of dogs with SIRS or sepsis, it was decreased, thereby suggesting it is a negative APP.^48^ Nevertheless, other causes for this discrepancy, such as hepatopathy or protein loss could not be ruled out.^48^ In our study, α_1_PI concentration was higher, albeit insignificantly so, after adjustment for multiple comparisons, in dogs with AP compared to controls, in agreement with studies of humans, warranting future studies in dogs.

Interestingly, in humans, α_2_AP and antithrombin inhibit pancreatic trypsin much faster (200‐fold) than does α_1_PI.^21^, ^49^ Although antithrombin depletion in AP might be attributed to its antithrombotic role, abnormal clotting times or thrombocytopenia were rare in our study. Thus, decreased ATA in AP more likely reflects its consumption as a PI, although viscoelastic tests were not performed, limiting our ability to assess antithrombin consumption in hypercoagulable states unaccompanied by increased D‐D concentration. Possibly, when severe pancreatic protease leakage overwhelms plasma antithrombin, α_1_PI might play a secondary PI role.^21^ This assumption is supported in our study by the negative correlation between α_1_PI concentration and the duration of historical clinical signs, suggesting that some α_1_PI consumption does occur in more prolonged AP. As a possible second line defense PI,^21^ α_1_PI seems a less useful early prognostic marker in dogs with AP, accounting for our observed lack of association between its concentration and death.

The αMGs are responsible for inhibition of trypsin and other serine proteases, and are depleted in humans and dogs with AP.^18^, ^23^ Nevertheless, the prognostic utility of serum α_2_MG concentration in humans with AP is disappointing, with sensitivity and specificity of 52%‐83% and 59%‐83%, respectively.^18^ The utility of αMGs in most clinical settings is limited by rapid αMG‐trypsin complex clearance.^50^, ^51^Additionally, in dogs with AP, α_2_MG concentration is not associated with outcome,^23^ suggesting limited utility as surrogate severity marker.^16^, ^42^ The α_2_MG concentrations, as measured using a commercial assay designed for humans, in our study were 100‐fold lower compared to previous results in dogs and humans,^23^, ^52^ likely indicating failure of this particular assay to accurately detect the canine protein, precluding investigation of group differences.

Pancreatic acinar injury leads to release of nuclear proteins (eg, histones), heat shock proteins and nuclear and mitochondrial DNA, inducing sterile inflammation through common immune sensors and inflammatory cytokines.^8^, ^53^, ^54^ This inflammatory storm results in SIRS, potentially damaging the lungs and kidneys, and resulting in MODS.^4^, ^55^ In a murine AP model, TNF‐α mRNA increased first, its concentrations positively associated with severity of inflammation.^56^ Tumor necrosis factor‐alpha has complex pro‐ and anti‐inflammatory activities in AP, depending on its binding, either to TNF‐α receptor 1 or 2.^56^, ^57^ Type‐1 receptor binding leads to programmed cell death, whereas type‐2 receptor binding induces cell survival and inflammation.^56^, ^57^ Both receptors also are shed as soluble forms, allowing circulating TNF‐α inactivation.^58^ With these complex binding and activity patterns, accurate measurement of TNF‐α using commercial assays may be difficult, because some only measure free TNFα, which has a short half‐life.^59^, ^60^ In our study, serum TNFα concentration was higher in dogs with AP compared to healthy controls, in contrast to previous findings,^17^ possibly because of assay differences. Nevertheless, its concentration was not associated with death or CAPS score, supporting a limited role for TNFα in prognostication of AP in dogs.

Interleukin‐6, a proinflammatory cytokine, contributes to lung injury and pancreatic necrosis in AP in humans.^61^, ^62^ It induces hepatic APP synthesis, including CRP.^63^ It increases within 24 hours of presentation, and is an early and sensitive prognostic marker of AP in humans.^15^, ^63^ In experimental AP in beagles, neutrophil‐derived IL‐6 mRNA peaks within 3 hours, in association with increased serum lipase activity and edematous changes in the pancreas.^64^ In another experimental AP study in dogs, IL‐6 administration prevented bacterial translocation,^65^ in the face of intestinal barrier function impairment, which occurs in dogs with AP.^66^ In our study, in agreement with previous findings,^17^ serum IL‐6 concentration was higher in the AP group compared to the control group, but was not associated with death, in contrast to findings in humans with AP.^15^, ^63^ Serum IL‐6 and CRP concentrations were positively correlated, supporting the role of IL‐6 in inducing hepatic APP synthesis.^63^ In agreement with an experimental pancreatitis model in dogs,^64^ day 0 serum IL‐6 concentration was correlated with higher day 1 serum cPLI concentration, linking lipase‐induced tissue damage and pancreatic lesions with inflammation in dogs with AP.^4^

Interleukin‐8 stimulates neutrophil activity.^10^ Its concentration is inconsistently associated with the severity of AP in humans, especially in later disease stages, peaking within 3 days from disease onset.^10^, ^67^ Serum IL‐8 concentration has not been reported previously in dogs with naturally occurring AP. Its day 0 and day 1 concentrations in our study did not differ between the AP and control groups. These findings however were likely influenced by the timing of sample collection, early during the disease course, because IL‐8 concentration possibly is associated with later complications.^14^

Interleukin‐2 is a marker of systemic lymphocyte activation in humans with AP.^68^, ^69^ Its soluble receptor (sIL‐2R) concentration peaks 1 day after onset of symptoms, remains high for at least 7 days, and is associated with pulmonary, renal and septic complications, and overall severity of AP in humans.^10^, ^69^ Preventing IL‐2‐mediated lymphocytic activation in mice with experimentally‐induced AP decreased systemic complications.^70^ Serum IL‐2 concentration in dogs with spontaneous AP has not been reported previously. In our study, its concentrations did not differ between AP and control groups, possibly suggesting a lesser role of IL‐2 in dogs with AP, although the results also might have been influenced by the assay used, which measures IL‐2 and not sIL‐2R.

C‐ reactive protein has been used as a prognostic marker in humans with AP,^13^, ^71^, ^72^ but because serum CRP concentration peaks 3 days after onset of symptoms, its clinical usefulness early during AP is limited.^10^ Similar to humans, previous studies showed that serum CRP concentration at presentation in dogs with AP increases, but only its concentration on days 3 or 4 significantly differs between survivors and nonsurvivors.^2^, ^39^ In our study, serum CRP concentration was measured relatively early during the course of AP, perhaps accounting for its lack of association with outcome. Interestingly, serum CRP concentration was positively and moderately correlated with both day 0 serum triglycerides and cholesterol concentrations. Hyperlipidemia in AP is associated with prolonged hospitalization, frequent MODS and increased fatality in humans.^73^, ^74^ Hypertriglyceridemia is also a therapeutic target in humans with AP, in whom heparin and insulin are used to lower serum triglyceride concentrations.^75^ Necrotic peripancreatic fat tissue may become an important source of inflammatory mediators, as manifested by increased concentrations of leptin, resistin, visfatin, and IL‐6,^17^ in support of our findings.

Although our findings might have prognostic utility, their clinical ramifications, mainly whether supplementation of depleted PIs is warranted, remain unclear. Although antithrombin treatment prevents development of AP and attenuates AP‐induced AKI in experimental AP models in mice and rats,^76^, ^77^ FFP administration was associated with death in an uncontrolled retrospective study of dogs with AP.^78^ In humans with AP, FFP administration did not affect outcome compared to negative controls,^79^ and FFP administered as a source of extrinsic antiproteases in severe AP failed to prevent a decrease in α_2_MG, or to increase patients' serum trypsin inhibition capacity.^44^ Nevertheless, a more precise approach, particularly relying on ATA, might better guide FFP treatment in dogs with AP, warranting prospective studies.

Our study had several limitations. First, its cohort size was limited, rendering it somewhat statistically underpowered. Indeed, several results that were borderline significant (ie, higher cPLI concentrations in nonsurvivors) might become significant in larger studies. Nevertheless, several present findings (eg, lack of association between cytokine concentrations and death, associations of ATA and CAPS score with death) are consistent with previous results,^25^, ^32^, ^33^ strengthening our conclusions. Second, the dynamic nature of inflammation in naturally occurring AP largely depends on the duration of clinical signs, as evident in differences between early and late presentations. This situation possibly affected our results, emphasizing the inherent limitations of clinical studies of naturally occurring diseases, where sampling times vary during the disease course. Third, with their similar clinical presentations, differentiating first AP episodes from acute flare‐ups of chronic, subclinical pancreatitis is not feasible clinically. Therefore, the AP group should more accurately be regarded as including dogs presenting with acute signs of pancreatitis, rather than dogs with AP. Fourth, a potential limitation may be caused by cPLI, PI, and cytokine decay during storage. Data from dogs and humans however indicate these analytes are stable for several years,^80^, ^81^, ^82^ suggesting a negligible or minimal storage duration effect on these deeply frozen samples. Lastly, analytes were measured only twice, within the first 2 days of hospitalization, precluding assessment of later changes, which may serve as future therapeutic targets. Nevertheless, early findings during the disease course, which were assessed in our study, are more relevant for the diagnosis and early prognostication of AP.

In conclusion, antithrombin is an important PI in dogs with AP. Its depletion is an important negative prognostic sign in such dogs, warranting prospective studies targeting its supplementation (ie, using FFP), which might improve survival. Although α_2_AP activity was lower in nonsurvivors, the differences between the outcome groups were small, and its activity did not differ between AP and control groups, suggesting that it is a less important PI and prognostic marker in dogs with AP. Conversely, α_1_PI seems to behave as a positive APP, possibly a second line PI, decreasing later during AP. Although inflammatory cytokines do increase in dogs with AP, supporting ongoing systemic inflammation, their concentrations were not associated with death, in agreement with previous findings.^17^ Finally, the CAPS scoring system has been prospectively validated, and higher CAPS scores were significantly associated with decreased ATA and increased inflammatory marker (CRP and IL‐6) and cPLI concentrations. Higher scores, alongside decreased ATA, are indicative of severe AP and a less favorable prognosis.

## CONFLICT OF INTEREST DECLARATION

Authors declare no conflict of interest.

## OFF‐LABEL ANTIMICROBIAL DECLARATION

Authors declare no off‐label use of antimicrobials.

## INSTITUTIONAL ANIMAL CARE AND USE COMMITTEE (IACUC) OR OTHER APPROVAL DECLARATION

This study was approved by the Veterinary Teaching Hospital and Koret School of Veterinary Medicine, Hebrew University, Institutional Ethics Committee (Reference number KSVM‐VTH/11_2014).

## HUMAN ETHICS APPROVAL DECLARATION

Authors declare human ethics approval was not needed for this study.

## Supporting information


**Appendix S1**. Severity scoring systems for acute pancreatitis in dogs
**Table S1**. Components and scores of the “organ score” clinical severity index for acute pancreatitis in dogs1

**Table S1**. Complete blood count results at presentation to the hospital of 31 dogs with acute pancreatitis.
**Table S2**. Serum chemistry results at presentation to the hospital of 31 dogs with acute pancreatitis.
