# Supplementary material for: Protease inhibitors, inflammatory markers, and their association with outcome in dogs with naturally occurring acute pancreatitis
Source: J Vet Intern Med. 2020 Sep 7;34(5):1801–12. doi: 10.1111/jvim.15895 (PMC7517856; doi:10.1111/jvim.15895)
Supplement: Supplementary file 1 — Appendix S1. Severity scoring systems for acute pancreatitis in dogs Table S1. Components and scores of the “organ score” clinical severity index for acute pancreatitis in dogs1 [file JVIM-34-1801-s001.pdf]

Appendix 1: Severity scoring systems for acute pancreatitis in dogs

Table 1: Components and scores of the "organ score" clinical severity index for acute pancreatitis in dogs[1]

system	parameters	score
**Endocrine**	No abnormalities	0
	Pre-existing diabetes mellitus	1
	Diabetic ketoacidosis	2
**Hepatic**	No abnormal changes	0
	≥ 2.5-fold increase (compared to upper limit of the reference interval) in ≥2 of serum activities of alkaline phosphatase, alanine transaminase and aspartate transaminase	1
	≥ 5-fold increase (compared to upper limit of the reference interval) ≥2 of serum activities of alkaline phosphatase, alanine transaminase and aspartate transaminase	2
	Extra-hepatic bile duct obstruction	3
**Renal**	No abnormalities	0
	Azotaemia [≤ 1.5-fold increase (compared with the upper limit of the reference interval) in serum urea and creatinine concentration]	1
	Anuria or azotaemia [≥ 1.5-fold increase (compared with the upper limit of the reference interval) of serum urea and creatinine concentrations]	2
**Hematopoietic**	No abnormalities	0
	WBCC ≥ 20.0×10^9 cells/L or ≤ $4.0\text{X}10^9$ cells/L and band neutrophils are ≤ 10% of neutrophils	1
	WBCC ≥ 20.0×10^9 cells/L or ≤ $4.0\text{X}10^9$ cells/L, neutrophil count ≤ $1.0\text{X}10^9$ cells/L, or if band neutrophils are ≥ 10% of neutrophils	2
	Laboratory evidence of hypercoagulability or hemostatic abnormalities	3
	Clinical evidence of DIC or bleeding diathesis	4
**Local complications**	No abnormalities	0
	Peritonitis extending beyond peri-pancreatic area	1
	Pseudocyst or other acute fluid accumulation	2
	Pancreatic abscess	3
**Cardiac**	No abnormalities	0
	<60 VPCs[6] / 24-hours period or heart rate > 180 bpm	1
	Paroxysmal or sustained ventricular tachycardia	2
**Respiratory**	No abnormalities	0
	Clinical evidence of dyspnoea or tachycardia (>40 breaths/min)	1
	Clinical evidence of pneumonia or ARDS	2
**Intestinal integrity**	No abnormalities	0
	Intestinal sounds not detected during >3 auscultations over a 24-hour period*	1
	Hematochezia, melena or regurgitation	2
	No food intake for > 3 days	3
	No food intake for > 3 and at least 2 of the following: hematochezia, melena and regurgitation	4
**Vascular forces**	No abnormalities	0
	Systolic arterial blood pressure < 60 or > 180 mm Hg or serum albumin concentration < 18 g/L	1
	Systolic arterial blood pressure < 60 or > 180 mm Hg and serum albumin concentration < 18 g/L	2

*, not routinely performed at the teaching hospital and this category was not scored in the present study; WBCC, white blood cell count; VPCs, ventricular premature complexes.

The canine acute pancreatitis severity (CAPS) scoring system[2] combines scores in several categories, including presence of systemic inflammatory response syndrome (SIRS), hemostatic disorders, increased serum creatinine (sCr) concentration (>1.6 mg/dL) and ionized hypocalcemia (Ca^{2+} <1.1 mmol/L). SIRS was diagnosed based on ≥2 of the following: heart rate >120 bpm; respiratory rate (RR) >20 breaths/min; rectal temperature >39.2°C or <38.1°C, and a white blood cell count <6000/µL or >16,000/µL. Hemostatic disorders were defined as presence of ≥1 of the following: marked thrombocytopenia (platelet count <63,000/µL) prolonged prothrombin time and prolonged activated partial thromboplastin time >25% of their upper reference limits.[2] These individual scores are applied into the following equation: 8 × (SIRS present, 1; SIRS absent, 0) + 3 × (hemostatic disorders present, 1, otherwise, 0) + 4 × (increased sCr, 1; otherwise, 0) + 3 × (ionized hypocalcemia present, 1; otherwise, 0).[2]

A simplified version of the CAPS scoring system (sCAPS) is based on the following formula: 3 × (RR ≥24 breaths/min, 1; otherwise, 0) + 3 × (hemostatic disorders present, 1; otherwise, 0) + 4 × (increased sCr 1; otherwise, 0) + 3 × (ionized hypocalcemia, 1; otherwise, 0).[2]

References (blinded)

1. Ruaux CG, Atwell RB. A severity score for spontaneous canine acute pancreatitis. Aust Vet J 1998;76:804-808.

2. Fabres V, Dossin O, Reif C, et al. Development and validation of a novel clinical scoring system for short-term prediction of death in dogs with acute pancreatitis. J Vet Intern Med 2019;33:499-507.
