# Supplementary material for: Protease inhibitors, inflammatory markers, and their association with outcome in dogs with naturally occurring acute pancreatitis
Source: J Vet Intern Med. 2020 Sep 7;34(5):1801–12. doi: 10.1111/jvim.15895 (PMC7517856; doi:10.1111/jvim.15895)
Supplement: Supplementary file 2 — Table S1. Complete blood count results at presentation to the hospital of 31 dogs with acute pancreatitis. Table S2. Serum chemistry results at presentation to the hospital of 31 dogs with acute pancreatitis. [file JVIM-34-1801-s002.pdf]

Supplementary Table 1: Complete blood count results at presentation to the hospital of 31 dogs with acute pancreatitis.

Analyte	n (%)<br>Median (range)	Within RI<br>n (%)	n (%) >RI	n (%) <RI	RI
Leukocytes ($10^3/mm^3$)	31 (100)<br>18.4 (6.7-57.9)	11 (35)	20 (65)	0 (0)	5.2-13.9
Neutrophils ($10^3/mm^3$)	31 (100)<br>15.7 (5.3-46.0)	6 (19)	25 (81)	0 (0)	3.9-8.0
Lymphocytes ($10^3/mm^3$)	31 (100)<br>1.17 (0.05-4.68)	16 (52)	1 (3)	14 (45)	1.3-4.1
Monocytes ($10^3/mm^3$)	31 (100)<br>1.05 (0.25-3.15)	23 (74)	8 (26)	0 (0)	0.2-1.1
Eosinophils ($10^3/mm^3$)	31 (100)<br>0.06 (0.0-0.36)	31 (100)	0 (0)	0 (0)	0.0-0.6
Basophils ($10^3/mm^3$)	31 (100)<br>0.03 (0.0-0.17)	29 (94)	2 (6)	0 (0)	0.0-0.1
Red blood cells ($10^6/mm^3$)	31 (100)<br>7.0 (3.0-9.6)	17 (55)	4 (13)	10 (32)	5.7-8.8
Hemoglobin (g/dL)	31 (100)<br>15.8 (5.2-23.6)	12 (39)	10 (32)	9 (29)	12.9-18.4
Hematocrit (%)	31 (10)<br>45.2 (16.2-68.2)	14 (45)	9 (29)	8 (26)	37.1-57.0
MCV (fL)	27 (87)<br>69.6 (54.0-77.0)	18 (67)	8 (29)	1 (4)	58.8-71.2
MCHC (g/dL)	26 (84)<br>33.9 (29.9-31.2)	16 (62)	7 (27)	3 (11)	31.0-36.2
RDW (%)	25 (81)<br>13.5 (11.2-31.1)	14 (56)	9 (36)	2 (8)	11.9-14.5
Platelets ($10^3/mm^3$)	31 (100)<br>340 (51-1704)	14 (45)	13 (42)	4 (13)	143.3-400.0
PDW ($10^3/mm^3$)	24 (77)<br>59.7 (15.4-88.5)	6 (26)	9 (37)	9 (37)	40.6-65.2
MPV (fL)	25 (81)<br>10.7 (7.2-22.3)	14 (56)	11 (44)	0 (0)	7.0-11.0

RI, reference interval; MCV, mean corpuscular volume; MCHC, mean corpuscular hemoglobin concentration; RDW, red blood cell distribution width; PDW, platelet volume distribution width; MPV, mean platelet volume.

Supplementary Table 2: Serum chemistry results at presentation to the hospital of 31 dogs with acute pancreatitis.

Analyte	n (%)<br>Median (range)	Within RI<br>n (%)	>RI<br>n (%)	>RI<br>n (%)	Reference interval
Alanine transaminase (U/L)	31 (100)<br>70 (24-1481)	15 (48)	16 (52)	0 (0)	19-67
Albumin (g/dL)	31 (100)<br>3.4 (2.3-4.9)	21 (68)	2 (6)	8 (26)	3.0-4.4
Alkaline phosphatase (U/L)	31 (100)<br>257 (19-6475)	10 (32)	20 (65)	1 (3)	21-170
Amylase (U/L)	30 (97)<br>2436 (368-33942)	10 (33)	20 (67)	0 (0)	103-1510
Aspartate transaminase (U/L)	26 (84)<br>59 (23-491)	8 (31)	18 (69)	0 (0)	19-42
Bilirubin (mg/dL)	31 (100)<br>0.15 (0.02-13.8)	21 (68)	10 (32)	0 (0)	0.0-0.2
β-hydroxybutyric acid (mmol/L)	5 (16)<br>6.9 (1.2-18.9)	0 (0)	5 (16)	0 (0)	0.0-0.7
Chloride (mmol/L)	25 (81)<br>104 (69-126)	13 (52)	1 (4)	11 (44)	104-118
Cholesterol (mg/dL)	29 (94)<br>273 (113-1818)	16 (55)	10 (35)	3 (10)	135-361
Creatine kinase (U/L)	20 (65)<br>241 (65-1141)	14 (70)	6 (30)	0 (0)	51-399
Creatinine (mg/dL)	31 (100)<br>0.8 (0.4-4.9)	23 (74)	8 (26)	0 (0)	0.3-1.2
γ-glutamyl transpeptidase (U/L)	23 (74)<br>5 (0.0-776)	15 (65)	8 (35)	0 (0)	0-6
Globulin (g/dL)	28 (90)<br>3.2 (2.2-6.25)	26 (93)	2 (7)	0 (0)	1.8-39
Glucose (mg/dL)	31 (100)<br>100 (55-1358)	20 (64)	7 (23)	4 (13)	64-123
Ionized calcium (mmol/L)	31 (100)<br>1.0 (0.74-1.36)	22 (71)	1 (3)	8 (26)	0.9-1.35
Lipase-DGGR (U/L)	24 (77)<br>1643 (294-13085)	0 (0)	24 (77)	0 (0)	0-107
Phosphorus (mg/dL)	26 (84)<br>4.6 (2.7-15.6)	18 (69)	7 (27)	1 (4)	3.0-6.2
Potassium (mmol/L)	31 (100)<br>4.1 (2.3-5.1)	26 (84)	0 (0)	5 (16)	3.6-5.3
Sodium (mmol/L)	26 (84)<br>142 (125-153)	16 (62)	0 (0)	10 (38)	140-154
Total calcium (mg/dL)	26 (84)<br>9.19 (6.28-11.9)	7 (27)	1 (4)	18 (69)	9.7-11.5
Total protein (g/dL)	28 (90)<br>6.6 (4.7-9.1)	21 (75)	4 (14)	3 (11)	5.4-7.6
Triglycerides (mg/dL)	20 (65)<br>120.7 (20-4021)	11 (55)	9 (45)	0 (0)	19-133
Urea (mg/dL)	29 (94)<br>37 (12-370)	19 (66)	10 (34)	0 (0)	10.7-53.5

RI, reference Interval; Lipase-DGGR, 1,2-o-dilauryl-rac-glycero glutaric acid-(6'-methylresorufin) ester, (DGGR)-lipase activity.
